# Comprehensive analysis based on tertiary lymphoid structures and tumor budding predicts survival rates in colorectal cancer patients with synchronous liver metastases

**DOI:** 10.3389/fimmu.2026.1776493

**Published:** 2026-05-12

**Authors:** Jianzhi Zhang, Hao Zhu, Long Qian, Mengjie Liang, Ji Miao, Haitao Zhu, Xiaofei Shen, Yonghuan Mao, Qiang Li

**Affiliations:** 1 Department of General Surgery, Nanjing Drum Tower Hospital, Affiliated Hospital of Medical School, Nanjing University, Nanjing, China; 2 Department of General Surgery, Affiliated Drum Tower Hospital, Nanjing University of Chinese Medicine, Nanjing, China; 3 Department of General Surgery, Nanjing Drum Tower Hospital Clinical College of Nanjing Medical University, Nanjing, China; 4 Colorectal Cancer Center, Department of General Surgery, Jiangsu Cancer Hospital, Cancer Research Institute, Cancer Hospital of Nanjing Medical University, Nanjing, China

**Keywords:** colorectal cancer liver metastasis, nomogram, prediction, tertiary lymphoid structures, tumor budding

## Abstract

**Background:**

Synchronous colorectal cancer liver metastasis (CRCLM) is closely associated with poor prognosis; however, the precise factors contributing to this adverse outcome remain uncertain. In the tumor microenvironment of CRCLM, tumor budding (TB) serves as a morphologic hallmark of invasive potential, while tertiary lymphoid structures (TLS) are indicative of organized anti-tumor immune contexture. This study aimed to integrate these biomarkers to predict the survival of patients with CRCLM.

**Methods:**

This multicenter retrospective study enrolled 107 patients with synchronous CRCLM who underwent curative resection for colorectal cancer. An external validation cohort including 30 patients with CRCLM were also employed. The density of TLS and the TB count were quantified via hematoxylin and eosin (H&E) staining and immunohistochemical (IHC) staining on primary tumor specimens. Relationships between clinicopathological data, overall survival (OS), and recurrence-free survival (RFS) were analyzed. The TLS/TB index was derived by integrating TLS and TB densities. The clinical role and prognostic value of the TLS/TB index in patients with CRCLM were analyzed, and a nomogram was constructed to predict the 3-year survival of patients.

**Results:**

High TLS density was significantly associated with prolonged overall survival (OS; p < 0.001) and recurrence-free survival (RFS; p< 0.001) in patients with CRCLM. Conversely, higher TB grade predicted a worse OS (p < 0.001) and RFS (p < 0.001). The TLS and TB exhibited a significant inverse correlation (r = -0.581, p < 0.001). The TLS/TB index demonstrated superior prognostic accuracy in the training cohort (AUC = 0.89) compared with histological differentiation (AUC = 0.70), TB grade (AUC = 0.85) and TLS density (AUC = 0.80), with consistent performance in the validation cohort. Calibration curves showed acceptable agreement between predicted and observed survival in both cohorts.

**Conclusion:**

These results demonstrated that tumor budding and tertiary lymphoid structures are inversely correlated in CRCLM. Furthermore, the TLS/TB-based nomogram may effectively enhanced the accuracy of prognosis prediction in patients with CRCLM, potentially facilitating the formulation of individualized treatment strategies.

## 1 Introduction

Colorectal cancer (CRC) is a malignancy characterized by high clinical aggressiveness and its development is closely associated with dietary and lifestyle factors. According to recent data, CRC accounts for an estimated 1.9 million new cases and 904,000 deaths globally, representing nearly one-tenth of all cancer cases and deaths. It is the third most common cancer and the second leading cause of cancer-related deaths worldwide. Currently, with the widespread adoption of colonoscopy screening, the 5-year survival rate of patients with early stage CRC is approximately 90% (1). However, for advanced or metastatic CRC, the 5-year survival rate drops to approximately 13% (1). Blood from the gastrointestinal tract enters the liver via the portal vein, facilitating the dissemination of colorectal cancer cells to the liver (2). Consequently, the liver is the most frequent site of metastasis in CRC (3). Approximately 50% of CRC patients develop liver metastases during the course of their disease (4). Despite advancements in surgical techniques and targeted therapies, the prognosis of patients with colorectal cancer liver metastasis (CRCLM) remains poor (5, 6). Understanding the specific factors influencing CRCLM patient outcomes and developing effective targeted therapies based on these factors is critically important for this patient population. Therefore, there is an urgent need for novel models to predict survival and recurrence in patients with liver metastases from colorectal cancer.

Numerous studies indicate that the tumor microenvironment (TME) plays a pivotal role in the progression of CRCLM (7, 8). The TME comprises cancer cells, non-cancerous cells (e.g., fibroblasts and immune cells), and extracellular matrix (ECM) (9, 10). The immune landscape within TME is intrinsically linked to cancer invasion and metastasis (11, 12). As tumors develop and invade surrounding tissues within the TME, they often manifest as single tumor cells or small clusters, a phenomenon known as tumor budding (TB). Tumor budding has long been associated with the epithelial-mesenchymal transition (EMT) and is considered a hallmark of invasive and migratory behavior in colorectal cancer (13, 14). Research demonstrates that TB is an independent adverse prognostic factor in CRC patients (15). Thus, TB serves as a marker for highly aggressive tumor subclones and is associated with poor clinical outcomes (16, 17).

In parallel, the host immune contexture plays a significant role in modulating tumor progression. Tertiary lymphoid structures (TLS) are ectopic lymphoid formations that develop in nonlymphoid organs during chronic inflammation, including cancer, infection, and autoimmune diseases (18). Within tumors, TLS represents the frontline of the anti-tumor immune response. They facilitate the rapid presentation of antigens by dendritic cells to T cells and provide a niche conducive to the activation, proliferation, and differentiation of T and B cells (19). The presence of intratumoral TLS is associated with a reduced risk of recurrence and improved survival in patients with various malignancies, including colorectal cancer (20, 21). Our group’s previous work similarly confirmed that TLS constitutes a significant protective factor against tumor progression (15, 22–25).

In summary, since the immune landscape of the TME correlates with the response to immunotherapy, gaining a deeper understanding of the TME immune landscape in CRCLM holds significant promise for these patients (26). Therefore, we aimed to develop a clinical model capable of reflecting the dynamic interplay within the tumor immune microenvironment to some degree. Currently, we posit that the presence of TB serves as a morphological indicator of a high invasive potential, while TLS reflects an organized host immune architecture within the microenvironment. Building upon our group’s prior research, the objective of this study was to jointly analyze TB and TLS as independent prognostic factors, leveraging their combined potential to predict outcomes in patients with CRCLM. Furthermore, this model aims to provide an integrative morphologic framework to assess the equilibrium between host immune organization and the invasive phenotype of the tumor. While acknowledging the inherent complexity of the tumor microenvironment, we hypothesize that the TLS/TB index may serve as a comprehensive tool for predicting survival and recurrence in CRCLM patients.

## 2 Materials and methods

### 2.1 Study design and patient cohort

**Figure 1:**
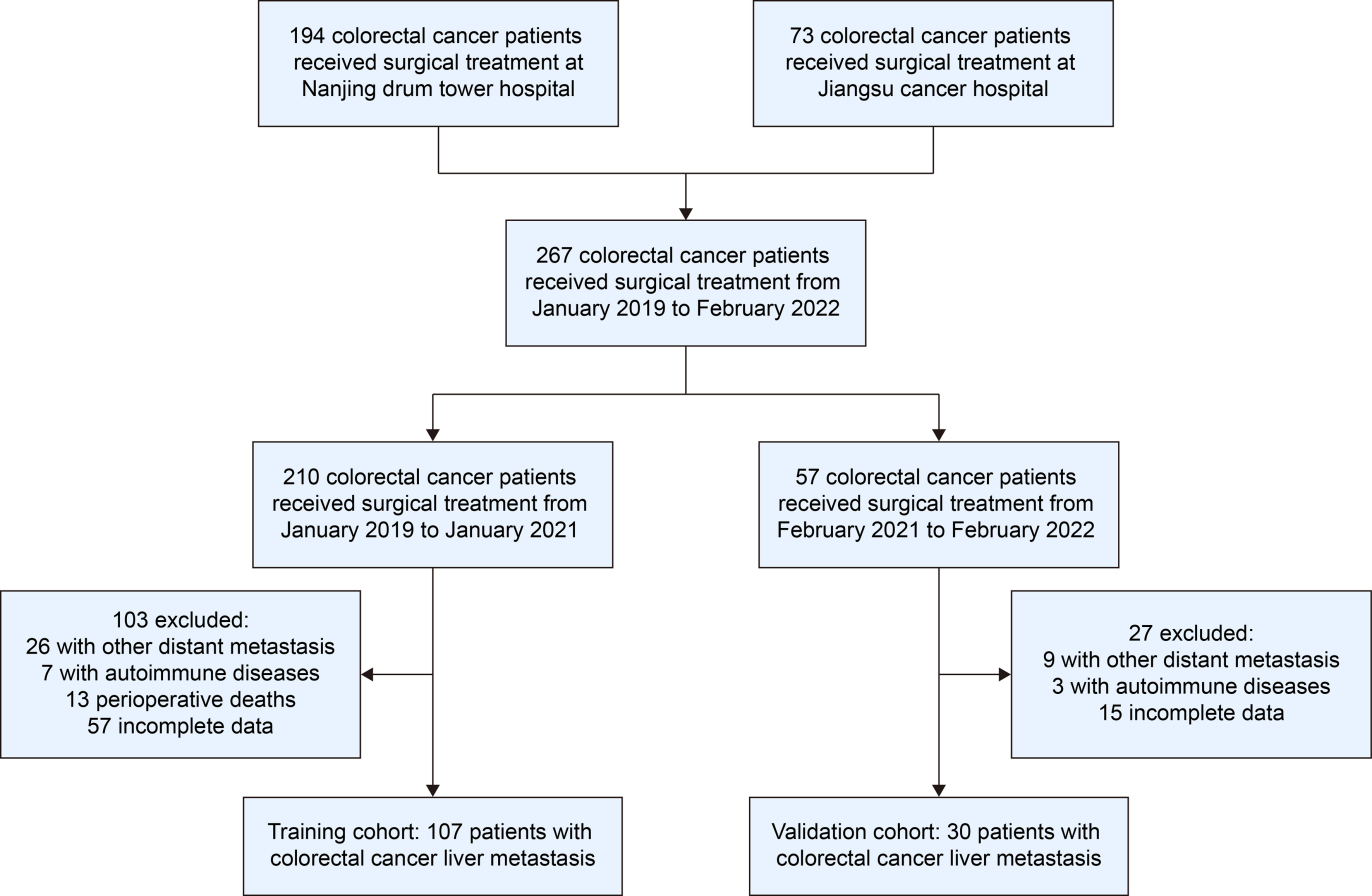
Flowchart of the study. A retrospective analysis was conducted on the clinical data of 194 patients with colorectal cancer liver metastases from Nanjing Drum Tower Hospital and 73 patients from Jiangsu Cancer Hospital. After applying strict inclusion and exclusion criteria, a total of 137 patients with colorectal cancer liver metastases were ultimately enrolled. Among them, 107 patients treated between January 2019 and January 2021 were assigned to the training cohort, while 30 patients treated between February 2021 and February 2022 were assigned to the validation cohort.

This study retrospectively analyzed 210 patients who underwent surgery at multiple medical centers between January 2019 and January 2021, all of whom underwent curative resection for colorectal cancer. Patients meeting the following criteria were included: age ≥ 18 years, curative resection for colorectal cancer, histopathologically confirmed colorectal cancer liver metastasis, and compliance with follow-up, while excluding those with synchronous metastases to other organs, a history of autoimmune disease requiring immunosuppressive medication, perioperative mortality, or incomplete pathological/missing follow-up data. Thus, all enrolled patients had synchronous liver metastases at the time of primary colorectal cancer diagnosis. Following stringent screening, 107 CRCLM patients were enrolled, with the study approved by the Ethics Committee of each participating medical center. Written informed consent for surgery and for the use of clinical data for research purposes was obtained from all patients. An independent external validation cohort comprising 30 patients with CRCLM was enrolled from February 2021 to February 2022 at the same institutions, using the same inclusion and exclusion criteria. The validation cohort was temporally separated from the training cohort to ensure independence, with the patient selection process detailed in **Figure 1**. All procedures performed in our study were in line with the strengthening the reporting of cohort, cross-sectional and case-control studies in surgery (STROCSS) criteria (27).

### 2.2 Observation index

Patient baseline information, including age, sex, tumor size, and tumor location, was collected. Postoperative pathological reports were analyzed to determine the tumor size, histological differentiation (well/moderate/poor), DNA mismatch repair (dMMR/pMMR), T stage (T1/T2/T3/T4), N stage (N0/N1/N2), surgical margin status (R0 vs. R1), number of liver metastases (≤3 vs>3), size of the largest liver metastasis (≤5cm vs>5cm), and preoperative treatment modality (none, Chemotherapy, or Chemoradiotherapy). Given the low 5-year survival rate of patients with CRCLM, all patients underwent postoperative follow-up for 3 years. Overall survival (OS) was defined as the time from the date of primary colorectal cancer surgery to the date of death from any cause or last follow-up. Recurrence-free survival (RFS) was defined as the time from the date of primary colorectal cancer surgery to the date of first documented disease recurrence (local or distant) or death from any cause, whichever occurred first. Patients who were alive and recurrence-free at the last follow-up were censored.

### 2.3 H&E and immunohistochemistry staining

Tumor specimens from primary colorectal cancer resections were processed into formalin-fixed, paraffin-embedded (FFPE) blocks. The FFPE blocks were sectioned at 4 μm thickness for H&E staining and IHC staining. TLS were defined as organized aggregates of lymphoid cells, typically consisting of a dense cluster of small lymphocytes with or without discernible germinal center formation, located within the tumor stroma or peritumoral region. Tumor-associated TLS were defined as those located within 7 mm of the tumor boundary (including the tumor area) (28). To further characterize the cellular composition of TLS in CRCLM, immunohistochemical (IHC) staining was performed on serial sections using the following antibodies: helper T cells (CD4; Abcam, ab133616, rabbit monoclonal, 1:200), cytotoxic T cells (CD8; Abcam, ab178089, rabbit monoclonal, 1:100), regulatory T cells (FOXP3; Abcam, ab20034, mouse monoclonal, 1:500), memory T cells (CD45RO; Abcam, mouse monoclonal, 1:1,000), B cells (CD20; Abcam, ab78237, rabbit monoclonal, 1:100), dendritic cells (CD11c; Abcam, ab52632, rabbit monoclonal, 1:500), natural killer cells (NCR1; Abcam, ab224703, rabbit monoclonal, 1:1,000), follicular dendritic cells (CD21; Abcam, ab75985, rabbit monoclonal, 1:100), macrophages (CD68; Wuhan Sevicebio Technology Co., Ltd, GB113150, rabbit monoclonal, 1:500), and tumor-associated neutrophils (CD15; Santa Cruz, SC-21702, mouse monoclonal, 1:100). These markers were used to confirm the lymphoid nature and maturity of TLS, with particular attention to CD20+ B-cell aggregates and CD21+ follicular dendritic cell networks, which indicate organized TLS with germinal center formation. The presence and location of TLS were independently assessed by two pathologists, with ICH-stained sections serving as complementary validation to the H&E-based evaluation. And to explore the relationship between TLS maturity and TB, TLS were further classified into three categories based on their maturation status on H&E-stained sections: Agg (aggregates of small lymphocytes without germinal center formation), FL1 (primary follicle-like structures with early germinal center formation), and FL2 (secondary follicle-like structures with well-defined germinal centers). For each patient, the percentage of each TLS category relative to the total TLS count was calculated. A continuous TLS maturity score was then derived using the formula: (percentage of Agg × 0) + (percentage of FL1 × 1) + (percentage of FL2 × 2). Receiver operating characteristic (ROC) curve analysis for overall survival was used to determine the optimal cutoff value for dichotomizing patients into low and high TLS maturity groups (cutoff for TLS maturity was found to be 0.371). This dichotomized maturity score was subsequently included as a covariate in survival analyses. Tumor budding (TB) was assessed using both H&E staining and IHC for cytokeratin 19 (CK19; antibody ab52625, dilution 1:200, Abcam). The assessment followed the International Tumor Budding Consensus Conference (ITBCC) 2016 recommendations. The use of CK19 IHC was justified based on its well-established role as a highly sensitive and specific marker for epithelial cells, including those in colorectal cancer. This approach enhances the detection of isolated single cells or small tumor cell clusters (≤4 cells) at the invasive front, which can sometimes be difficult to distinguish definitively from stromal or inflammatory cells on H&E staining alone, thereby improving the accuracy and reproducibility of TB scoring (29). To integrate the H&E and IHC data for TB assessment, the following procedure was applied: Initially, the region of highest budding intensity at the invasive front was identified under low-power magnification on H&E-stained slides. Subsequently, consecutive sections stained for CK19 were examined in the corresponding histologic area. The TB count was ultimately performed at ×200 magnification, preferentially using the CK19-stained slides to ensure precise identification of budding foci. In cases where IHC was unavailable or equivocal, the H&E-based evaluation was retained. This integrated approach ensured that the final TB quantification leveraged the enhanced detection capability of CK19 IHC while maintaining consistency across all samples (30). TB was categorized into three grades according to ITBCC criteria: low grade (0–4 buds), intermediate grade (5–9 buds), and high grade (≥10 buds). Both TLS and TB were quantitatively analyzed in colorectal cancer tissues. The assessment of both TLS and TB was performed independently by two pathologists. Prior to formal evaluation, they underwent a calibration session using a training set of slides to align their scoring criteria. The interobserver agreement for the continuous variables (TLS density and TB count) was quantitatively assessed using the Intraclass Correlation Coefficient (ICC). The analysis demonstrated excellent agreement, with an ICC of 0.93 (p<0.001, 95% CI: 0.90-0.95) for TLS density and an ICC of 0.91 (p<0.001, 95% CI: 0.87-0.94) for TB count. For the few instances where initial assessments differed, a predefined consensus procedure was followed: the discrepant cases were reviewed by a third senior pathologist, whose judgment served as the final arbiter.

### 2.4 Statistical analysis

Statistical analyses were performed using R Studio (version 3.6.3) and SPSS (version 27.0). For comparisons of clinicopathological characteristics between groups, categorical variables were analyzed using the χ² test or Fisher’s exact test, as appropriate. Continuous variables were compared using Student’s t-test or ANOVA. Survival outcomes, including overall survival (OS) and recurrence-free survival (RFS), were estimated using the Kaplan-Meier method, and differences between groups were assessed using the log-rank test. To evaluate the correlations among different variables, Pearson correlation analysis was applied for normally distributed data, and Spearman rank correlation analysis was used for non-normally distributed data. Variables showing significant results in univariate analysis were further analyzed using multivariate Cox regression. For model evaluation, receiver operating characteristic (ROC) curves were generated using the pROC package in R to assess the discriminatory ability of the TLS/TB index, TLS density, TB grade, and histological differentiation for 1−year and 3−year overall survival. The area under the curve (AUC) was calculated with 95% confidence intervals. Calibration curves were constructed using the rms package with 1,000 bootstrap resamples to reduce overfitting, and the agreement between predicted and observed survival probabilities was evaluated visually. Decision curve analysis was performed using the rmda package to quantify the net clinical benefit of the nomogram across a range of threshold probabilities. R software was used for nomogram construction, calibration curve analysis, and decision curve analysis. Statistical significance was set at P < 0.05.

## 3 Results

### 3.1 Comparison of clinicopathological characteristics of CRCLM patients between training cohort and validation cohort

We retrospectively analyzed 107 CRCLM patients and these patients were used as the training cohort. 30 CRCLM patients were used as an external validation set. There was no significant difference between the training and validation cohorts in demographic and clinical characteristics (**Table 1**).

**Table 1:** Comparison between training cohort and validation cohort of clinicopathological characteristics of CRCLM.

Variables	Training cohort (*n*=107)	Validation cohort (*n*=30)	Statistic	*P*
Age, n (%)			χ²=0.00	0.992
≥55	75 (70.09)	21 (70.00)		
<55	32 (29.91)	9 (30.00)		
Sex, n (%)			χ²=2.16	0.142
male	28 (26.17)	4 (13.33)		
female	79 (73.83)	26 (86.67)		
Tumor size			χ²=0.39	0.535
≤2cm	61 (57.01)	19 (63.33)		
>2cm	46 (42.99)	11 (36.67)		
Tumor site, n (%)			χ²=0.67	0.715
Left	41 (38.32)	12 (40.00)		
Right	37 (34.58)	12 (40.00)		
Rectal	29 (27.10)	6 (20.00)		
LN Metastasis, n (%)			χ²=0.05	0.830
Negative	23 (21.50)	7 (23.33)		
Positive	84 (78.50)	23 (76.67)		
T stage, n (%)			-	0.742
I	3 (2.80)	1 (3.33)		
II	6 (5.61)	3 (10.00)		
III	80 (74.77)	21 (70.00)		
IV	18 (16.82)	5 (16.67)		
Ki-67, n (%)			χ²=0.96	0.328
≤60%	36 (33.64)	13 (43.33)		
>60%	71 (66.36)	17 (56.67)		
Differentiation, n (%)			χ²=0.03	0.867
Well, Moderate	66 (61.68)	18 (60.00)		
Poor	41 (38.32)	12 (40.00)		
TLS density, n (%)			χ²=0.00	0.964
Low	54 (50.47)	15 (50.00)		
High	53 (49.53)	15 (50.00)		
Surgical Margin, n (%)			χ²=0.43	0.512
Negative	102 (95.33)	30 (100.00)		
Positive	5 (4.67)	0 (0.00)		
Vasculature Violations, n (%)			χ²=0.93	0.336
Negative	50 (46.73)	17 (56.67)		
Positive	57 (53.27)	13 (43.33)		
Perineural Invasion, n (%)			χ²=2.17	0.140
Negative	41 (38.32)	16 (53.33)		
Positive	66 (61.68)	14 (46.67)		
MMR, n (%)			χ²=0.00	1.000
pMMR	101 (94.39)	28 (93.33)		
dMMR	6 (5.61)	2 (6.67)		
TB, n (%)			χ²=1.76	0.414
Low	34 (31.78)	13 (43.33)		
Moderate	53 (49.53)	11 (36.67)		
High	20 (18.69)	6 (20.00)		
Neoadjuvant therapy type, n (%)			χ²=0.18	0.914
None	52 (48.60)	14 (46.67)		
Chemotherapy	35 (32.71)	11 (36.67)		
Chemoradiotherapy	20 (18.69)	5 (16.67)		
Metastasis size, n (%)			χ²=0.00	0.964
≤5cm	53 (49.53)	15 (50.00)		
>5cm	54 (50.47)	15 (50.00)		
Metastasis number, n (%)			χ²=0.70	0.404
≤3	55 (51.40)	18 (60.00)		
>3	52 (48.60)	12 (40.00)		
TLS maturity			χ²=1.53	0.216
Low	60 (56.07)	13 (43.33)		
High	47 (43.93)	17 (56.67)		

### 3.2 Correlation of TLSs and TBs with Prognosis and relationships between the TLSs and TBs in CRCLM

**Figure 2:**
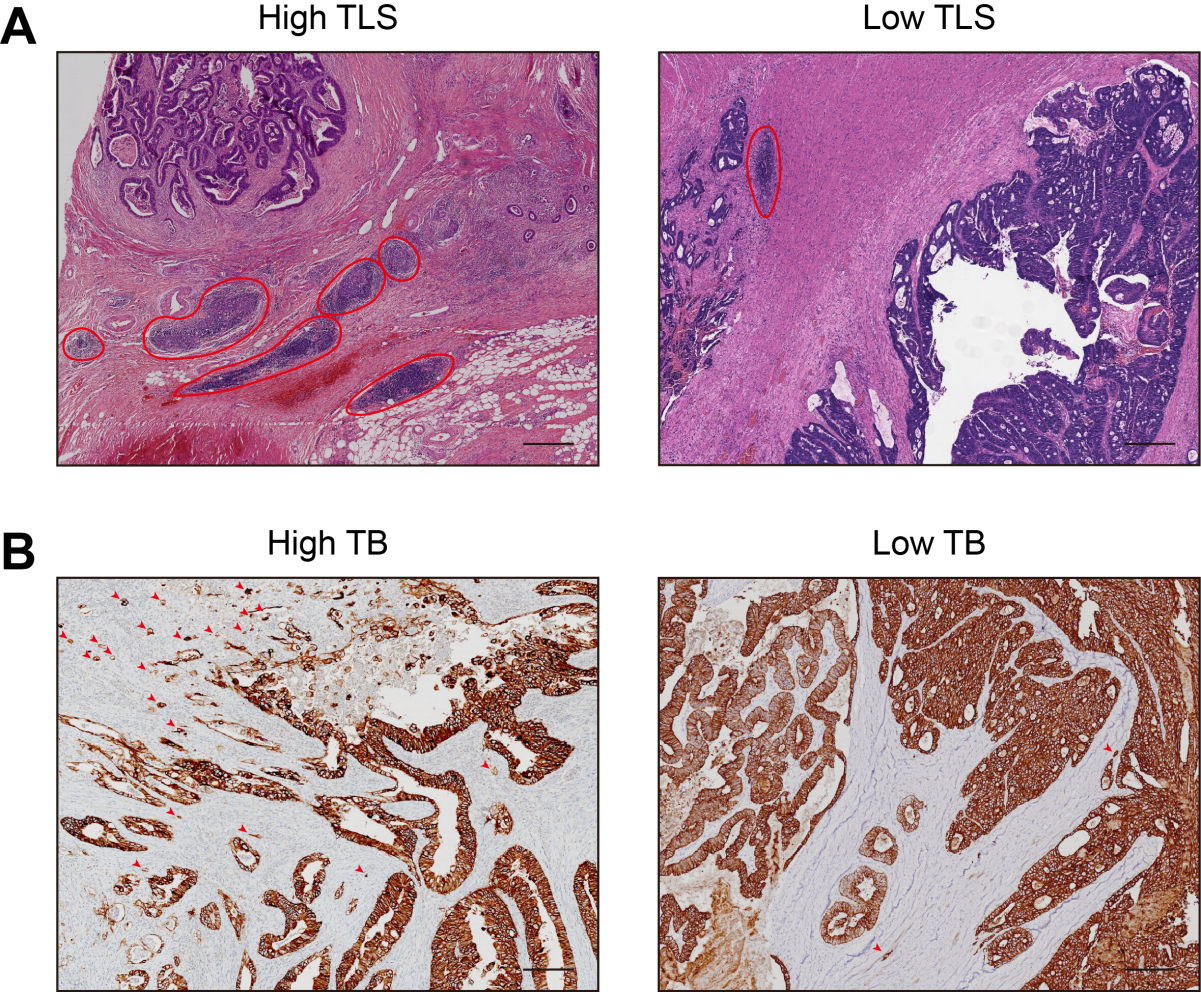
H&E and IHC sections showing TLS and TB. **(A)** H&E‐stained images of TLS in primary colorectal cancer tissues from patients with CRCLM (scale bar = 400 μm). **(B)** IHC‐stained images of TB in primary colorectal cancer tissues from patients with CRCLM (scale bar = 200 μm). TB, tumor budding; TLS, tertiary lymphoid structure; H&E, Hematoxylin and eosin; IHC, Immunohistochemistry; CRCLM, colorectal cancer liver metastases.

**Figure 3:**
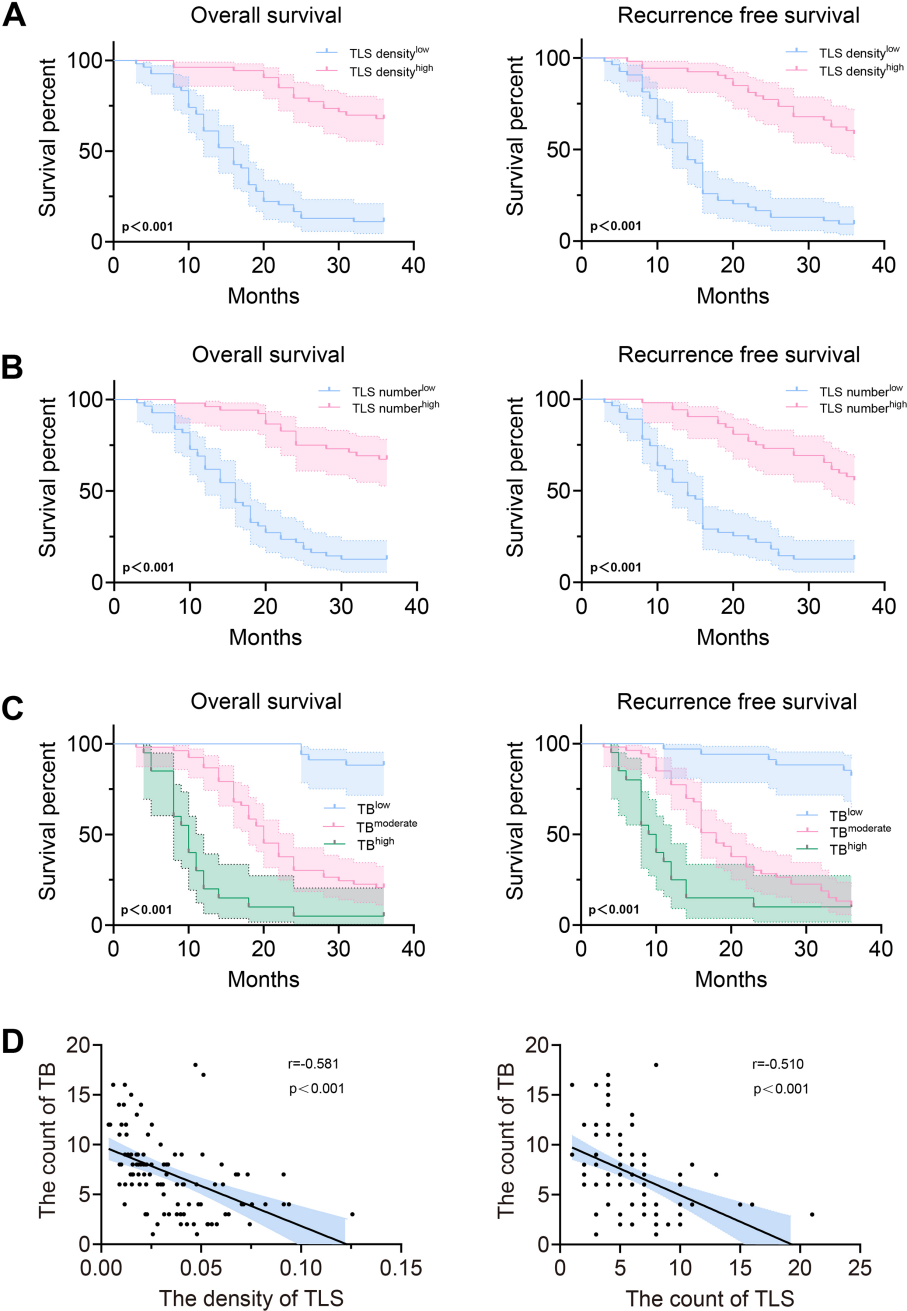
Correlation of TLSs and TBs with prognosis. **(A)** The Kaplan-Meier curves demonstrate differences in OS and RFS across varying TLS density. **(B)** The Kaplan-Meier curves demonstrate differences in OS and RFS across varying TLS numbers. **(C)** Kaplan-Meier curves demonstrate differences in OS and RFS across varying TB grade. **(D)** Scatter plots indicated the relationship between TLS and TB. OS, overall survival; RFS, recurrence-free survival; TLS, tertiary lymphoid structure; TB, tumor budding.

**Figure 4:**
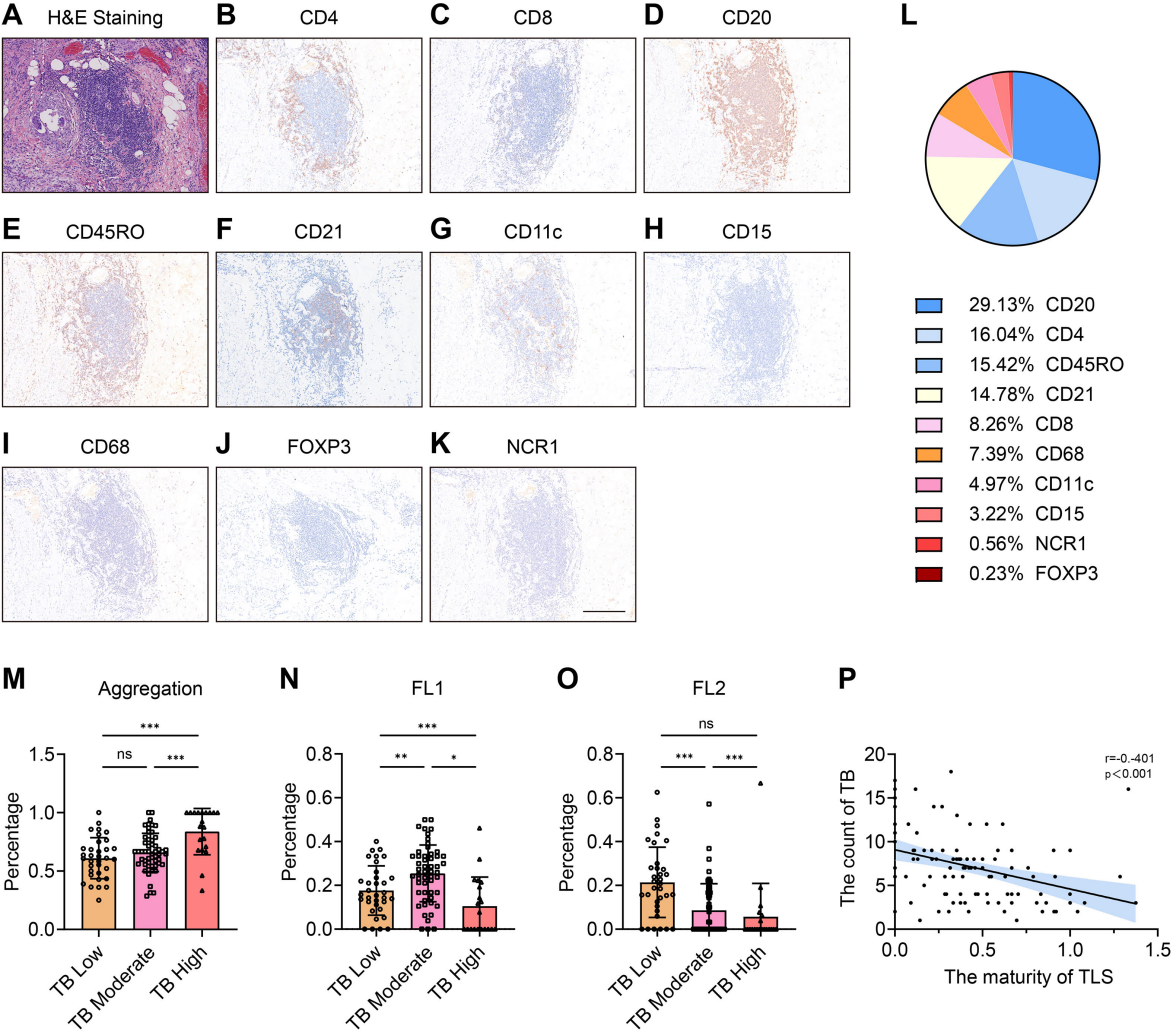
H&E sections showing TLS and different immune cells in TLS. **(A)** Representative figure of H&E slides to show TLS. **(B-K)** IHC results showed the different immune cells, which formed the TLS, including **(B)** CD4 + T cells, **(C)** CD8 + T cells, **(D)** CD20 + B cells, **(E)** CD45RO+ memory T cells, **(F)** CD21 + follicular dendritic cells, **(G)** CD11c + dendritic cells, **(H)** CD15 + granulocytes, **(I)** CD68 + macrophages, **(J)** FOXP3 + Treg cells, and **(K)** NCR1 + natural killer cells. **(L)** Pie chart figure showed the distribution of various immune cells forming the TLS. **(M–O)** column chart showed different TLS maturity status between different TB grade. **(P)** Scatter plots indicated the relationship between TLS maturity and TB counts. TB, tumor budding; TLS, tertiary lymphoid structure; H&E, Hematoxylin and eosin; IHC, Immunohistochemistry. *: p < 0.05; **: p < 0.01; ***: p < 0.001.

Representative H&E-stained images of TLS and TB in primary colorectal cancer tissues from patients with CRCLM are shown in **Figures 2A, B**, respectively. Based on histopathological quantification through H&E and IHC, TLS was stratified into low-density/high-density groups and low-count/high-count groups using median cutoffs. Using Kaplan-Meier analysis with the log-rank test, prognostic analysis revealed that both TLS high-density and high-count TLS groups exhibited superior overall survival (OS) and recurrence-free survival (RFS) (**Figures 3A, B**), whereas higher tumor budding grade correlated with worse clinical outcomes (**Figure 3C**). Based on these findings, we hypothesized potential interactions between TLS and TB. Subsequent correlation analysis demonstrated significant negative correlations between the TLS parameters (maturity/density/count) and TB counts (**Figure 3D**). We further explored the relationship between TB and TLS maturity subtypes (Agg, FL1, FL2). As shown in **Figure 4**, the distribution of TLS maturity subtypes differed significantly across TB grades: higher TB grades were associated with a higher proportion of Agg (immature aggregates) and a lower proportion of FL2 (mature TLS with germinal centers). The TLS maturity score, calculated as (percentage of Agg × 0) + (percentage of FL1 × 1) + (percentage of FL2 × 2), also showed a significant negative correlation with TB count (r = -0.401, p < 0.01).

### 3.3 Clinical significance of the TLS/TB index in CRCLM patients

Current research on TLS has advanced rapidly, with prevailing evidence suggesting that TLS reflects host immune defense mechanisms and predicts the prognosis of patients with CRCLM. Conversely, TB, as a manifestation of epithelial-mesenchymal transition (EMT), is widely regarded as an indicator of tumor aggressiveness, and higher TB grades correlate with increased invasiveness and poorer clinical outcomes. While various biomarkers have been explored in CRCLM, limited studies have focused on an integrative metric that specifically combines the quantitative density of TLS and TB. We therefore proposed a novel TLS/TB index to evaluate the potential balance between tumor-cell invasive patterns and organized lymphoid formations. To address this, we propose a novel TLS/TB index designed to quantify the balance between local immune response and tumor invasiveness, with the goal of improving prognostic stratification for CRCLM patients.

**Figure 5:**
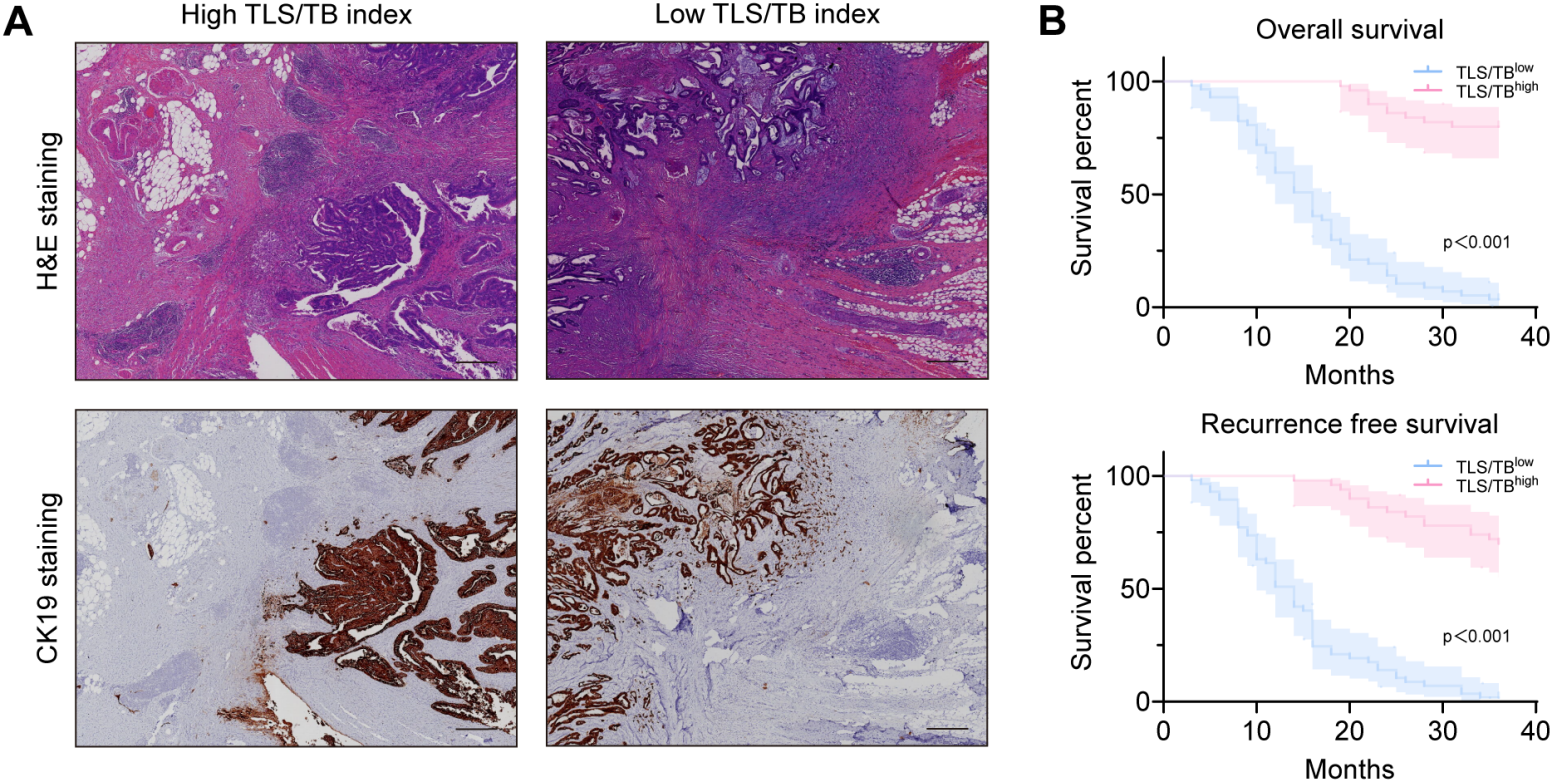
Correlation of the TLS/TB index with prognosis. **(A)** Co‐localization H&E-stained and IHC-stained images of high and low TLS/TB index in primary colorectal cancer tissues from patients with CRCLM. (scale bar = 400 μm). **(B)** The Kaplan-Meier curves demonstrate differences in OS and RFS across TLS/TB index. OS, overall survival; RFS, recurrence-free survival; TLS, tertiary lymphoid structure; TB, tumor budding; H&E, Hematoxylin and eosin; IHC, Immunohistochemistry.

Correlation analysis revealed a stronger inverse association between TLS density and TB count (r = -0.581, p < 0.001) than between TLS and TB counts or between TLS maturity and TB count. To standardize units (given a fixed microscopic field area of 0.785 mm² for TB quantification), both TLS and TB metrics were converted to density measurements. Specifically, since TB was assessed within a fixed microscopic field area of 0.785 mm², TB counts were converted to TB density (number/mm²) by dividing the count by the area of the field. The TLS/TB index was then defined as the ratio of TLS density (number/mm²) to TB density (number/mm²). This ratio is a dimensionless quantity. Using the median cutoff value (0.0041), patients were stratified into high and low TLS/TB groups (representative H&E/IHC images in **Figure 5A**). Significant intergroup differences were observed in tumor location (p = 0.046), Liver metastasis number (p=0.015) and histological differentiation (p < 0.001). Kaplan-Meier analysis (**Figure 5B**) demonstrated superior survival and reduced recurrence in the high TLS/TB index group. Furthermore, the high TLS/TB index group was more frequently associated with left-sided colon and rectal cancers, exhibited better histological differentiation and less Liver metastasis number (**Table 2**). Univariate analysis (using a threshold of p < 0.05 for inclusion in multivariate analysis) was performed. Prior to conducting multivariate analysis, we assessed the variance inflation factors (VIFs) to evaluate potential multicollinearity among TLS density, TB grade, and the TLS/TB index (31). The VIF values were 2.257 for TLS density, 2.039 for TB grade, and 3.470 for the TLS/TB index, all well below the common threshold of 5, indicating that multicollinearity was not a critical concern in our model. Subsequently, multivariate analysis identified histological differentiation, TLS density, TB grade, and the TLS/TB index as independent prognostic factors for patients with advanced colorectal cancer liver metastases (**Table 3**). Interestingly, for RFS, only TB grade was identified as an independent predictor, whereas TLS density did not retain independent significance (**Table 4**). This divergence may reflect the differential roles of these features in the tumor microenvironment. High TLS density likely contributes to a more robust antitumor immune response, which may predominantly impact long-term survival by controlling disease progression, rather than directly preventing early recurrence events that are more strongly driven by tumor invasiveness. Conversely, higher TB grade, reflecting enhanced invasive and migratory capacity, directly increases the risk of early recurrence and also contributes to poorer overall survival.

**Table 2:** Clinicopathological characteristics between TLS/TB^high^ and TLS/TB^low^ group of CRCLM patients.

Variables	TLS/TB index		*P*
	Low(n = 57)	High(n = 50)	
Age, n (%)			0.687
<55	18 (31.58)	14 (28.00)	
≥55	39 (68.42)	36 (72.00)	
Gender, n (%)			0.686
Male	43 (75.44)	36 (72.00)	
Female	14 (24.56)	14 (28.00)	
Tumor Site, n (%)			**0.046**
Right	28 (49.12)	13 (26.00)	
Left	17 (29.82)	20 (40.00)	
Rectum	12 (21.05)	17 (34.00)	
Tumor Size, n (%)			0.329
<5cm	30 (52.63)	31 (62.00)	
≥5cm	27 (47.34)	19 (38.00)	
Perineural Invasion, n (%)			0.737
Negative	21 (36.84)	20 (40.00)	
Positive	36 (63.16)	30 (60.00)	
Vasculature Violations, n (%)			0.525
Negative	25 (43.86)	25 (50.00)	
Positive	32 (56.14)	25 (50.00)	
LN Metastasis, n (%)			0.288
Negative	10 (17.54)	13 (26.00)	
Positive	47 (82.46)	37 (74.00)	
Differentiation,n (%)			<0.001
Well, Moderate	26 (45.61)	40 (80.00)	
Poor	31 (54.39)	10 (20.00)	
MMR, n (%)			1.000
dMMR	54 (94.39)	47 (94.00)	
pMMR	6 (5.61)	3 (6.00)	
T stage, n (%)			0.510
T1	2 (3.51)	1 (2.00)	
T2	2 (3.51)	4 (8.00)	
T3	41 (71.93)	39 (78.00)	
T4	12 (21.05)	6 (12.00)	
Ki-67, n (%)			0.455
≤60%	21 (36.84)	15 (30.00)	
>60%	36 (63.16)	35 (70.00)	
Neoadjuvant therapy type, n (%)			0.793
None	20(50.88)	23 (46.00)	
Chemotherapy	17(29.82)	18(36.00)	
Chemoradiotherapy	11(19.30)	9(18.00)	
Metastasis size, n (%)			0.210
≤5cm	25 (43.86)	28 (56.00)	
>5cm	32 (56.14)	22 (44.00)	
Metastasis number, n (%)			**0.015**
≤3	23 (40.35)	32 (64.00)	
>3	34 (59.65)	18 (36.00)	

p-values less than 0.05 are shown in bold.

**Table 3:** Univariate and multivariate analysis for OS using Cox regression in CRCLM.

Characteristics	Univariate analysis		Multivariate analysis	
	Hazard ratio (95% CI)	P-value	Hazard ratio (95% CI)	P-value
Age		0.614		
≥55	Reference			
<55	1.15 (0.68 ~ 1.94)			
Sex		0.546		
male	1.00 (Reference)			
female	1.19 (0.68 ~ 2.09)			
Tumor site				
Right	1.00 (Reference)		1.00 (Reference)	
Left	0.69 (0.40 ~ 1.19)	0.180	0.90 (0.49 ~ 1.64)	0.725
Rectal	0.45 (0.23 ~ 0.86)	**0.017**	0.62 (0.30 ~ 1.28)	0.196
Tumor size		0.357		
<2cm	1.00 (Reference)			
≥2cm	1.26 (0.77 ~ 2.05)			
Differentiation		**<0.001**		**<0.001**
Well, Moderate	1.00 (Reference)		1.00 (Reference)	
Poor	3.45 (2.09 ~ 5.69)		2.70 (1.52 ~ 4.78)	
Perineural Invasion		0.160		
Negative	1.00 (Reference)			
Positive	1.45 (0.86 ~ 2.44)			
Vasculature Violations		0.286		
Negative	1.00 (Reference)			
Positive	1.31 (0.80 ~ 2.14)			
LN Metastasis		0.057		
Negative	1.00 (Reference)			
Positive	1.98 (0.98 ~ 4.01)			
MMR status		0.441		
dMMR	1.00 (Reference)			
pMMR	1.49 (0.54 ~ 4.10)			
T stage		0.768		
I	1.00 (Reference)			
II	0.12 (0.01 ~ 1.20)	0.072		
III	0.62 (0.19 ~ 2.00)	0.426		
IV	0.75 (0.21 ~ 2.65)	0.660		
TB		**<0.001**		
Low	1.00 (Reference)		1.00 (Reference)	
Moderate	11.92 (4.25 ~ 33.46)	**<0.001**	4.62 (1.46 ~ 14.55)	**0.009**
High	44.12 (14.64 ~132.96)	**<0.001**	7.95 (2.23 ~ 28.36)	**0.001**
TLS density		**<0.001**		**0.030**
Low	1.00 (Reference)		1.00 (Reference)	
High	0.15 (0.09 ~ 0.27)		0.47 (0.24 ~ 0.93)	
TLS/TB index		**<0.001**		**0.003**
Low	1.00 (Reference)		1.00 (Reference)	
High	0.07 (0.03 ~ 0.14)		0.25 (0.10 ~ 0.62)	
Ki67				
≥60%	1.00 (Reference)			
<60%	1.17 (0.69 ~ 1.99)			
Neoadjuvant therapy type, n (%)		0.733		
None	1.00 (Reference)			
Chemotherapy	0.81 (0.47 ~ 1.41)	0.453		
Chemoradiotherapy	0.85 (0.43 ~ 1.68)	0.648		
Metastasis size, n (%)		0.455		
≤5cm	1.00 (Reference)			
>5cm	1.19 (0.75 ~ 1.90)			
Metastasis number, n (%)		**0.033**		0.366
≤3	1.00 (Reference)		1.00 (Reference)	
>3	1.71 (1.05 ~ 2.79)		1.30 (0.73 ~ 2.32)	
TLS maturity		**0.046**		0.618
Low	1.00 (Reference)		1.00 (Reference)	
High	0.60 (0.36 ~ 0.99)		0.87 (0.51 ~ 1.49)	

p-values less than 0.05 are shown in bold.

**Table 4:** Univariate and multivariate analysis for RFS using Cox regression in CRCLM.

Characteristics	Univariate analysis		Multivariate analysis	
	Hazard ratio (95% CI)	P-value	Hazard ratio (95% CI)	P-value
Age		0.614		
<55	1.00 (Reference)			
≥55	1.34 (0.82 ~ 2.20)			
Sex		0.546		
male	1.00 (Reference)			
female	1.13 (0.66 ~ 1.94)			
Tumor site				
Right	1.00 (Reference)		1.00 (Reference)	
Left	0.67 (0.39 ~ 1.13)	0.131	0.73 (0.41 ~ 1.30)	0.279
Rectal	0.42 (0.22 ~ 0.78)	**0.006**	0.55 (0.27 ~ 1.12)	0.098
Tumor size		0.460		
<2cm	1.00 (Reference)			
≥2cm	1.19 (0.75 ~ 1.91)			
Differentiation		**<0.001**		**0.002**
Well, Moderate	1.00 (Reference)		1.00 (Reference)	
Poor	3.54 (2.19 ~ 5.73)		2.50 (1.41 ~ 4.45)	
Perineural Invasion		0.142		
Negative	1.00 (Reference)			
Positive	1.45 (0.88 ~ 2.39)			
Vasculature Violations		0.150		
Negative	1.00 (Reference)			
Positive	1.42 (0.88 ~ 2.27)			
LN Metastasis		**0.031**		0.051
Negative	1.00 (Reference)		1.00 (Reference)	
Positive	2.09 (1.07 ~ 4.09)		2.14 (1.00 ~ 4.58)	
MMR status		0.606		
dMMR	1.00 (Reference)			
pMMR	1.30 (0.48 ~ 3.58)			
T stage		0.768		
I	1.00 (Reference)			
II	0.10 (0.01 ~ 1.00)	0.050		
III	0.61 (0.19 ~ 1.97)	0.409		
IV	0.78 (0.22 ~ 2.72)	0.696		
TB		**<0.001**		
Low	1.00 (Reference)		1.00 (Reference)	
Moderate	12.50 (4.92 ~ 31.77)	**<0.001**	6.46 (2.24 ~ 18.64)	**<0.001**
High	38.48 (13.96 ~ 106.05)	**<0.001**	12.86 (3.72 ~ 44.44)	**<0.001**
TLS density		**<0.001**		0.128
Low	1.00 (Reference)		1.00 (Reference)	
High	0.09 (0.05 ~ 0.16)		0.56 (0.27 ~ 1.18)	
TLS/TB index		**<0.001**		**0.022**
Low	1.00 (Reference)		1.00 (Reference)	
High	0.07 (0.03 ~ 0.14)		0.36 (0.15 ~ 0.86)	
Ki67				
<60%	1.00 (Reference)			
≥60%	1.06 (0.65 ~ 1.75)			
Neoadjuvant therapy type, n (%)				
None	1.00 (Reference)			
Chemotherapy	0.99 (0.59 ~ 1.65)	0.961		
Chemoradiotherapy	0.81 (0.41 ~ 1.59)	0.541		
Metastasis size, n (%)		0.849		
≤5cm	1.00 (Reference)			
>5cm	1.05 (0.64 ~ 1.71)			
Metastasis number, n (%)		**0.029**		0.409
≤3	1.00 (Reference)		1.00 (Reference)	
>3	1.69 (1.06 ~ 2.70)		1.26 (0.73 ~ 2.15)	
TLS maturity		**0.046**		0.901
Low	1.00 (Reference)		1.00 (Reference)	
High	0.61 (0.38 ~ 0.99)		0.97 (0.58 ~ 1.62)	

p-values less than 0.05 are shown in bold.

### 3.4 Construction and validation of the nomogram

**Figure 6:**
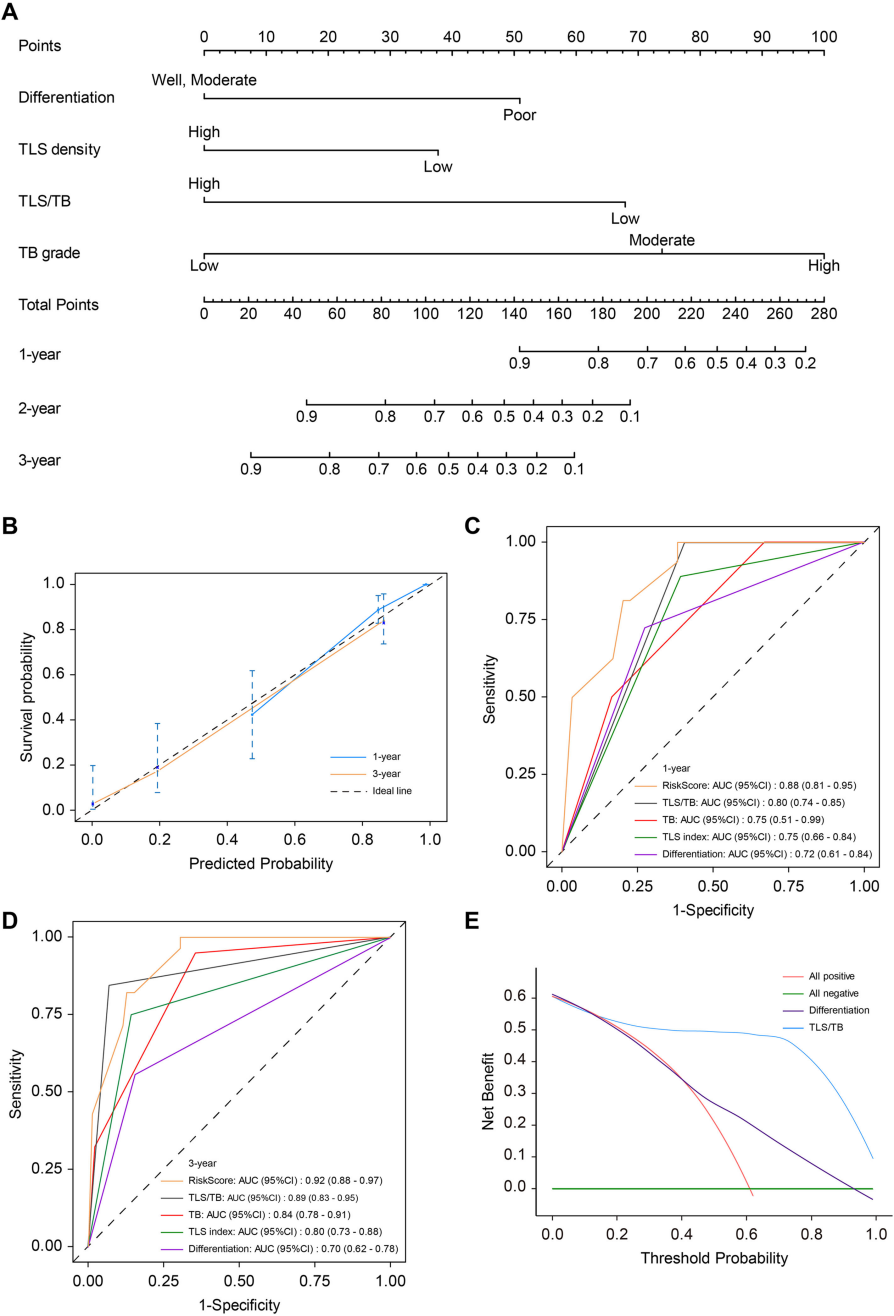
Construction and validation of the nomogram for CRCLM. **(A)** The nomogram to predict 1-year, 2-year and 3-year survival probability for CRCLM. **(B)** The calibration curves of 1-year and 3-year survival probability in CRCLM. **(C)** The ROC curve of 1-year survival probability in CRCLM, comparing the risk-score model with Differentiation, TLS index, TB and TLS/TB index alone. **(D)** The ROC curve of 3-year survival probability in CRCLM, comparing the risk-score model with Differentiation, TLS index, TB and TLS/TB index alone. **(E)** Decision curve analysis based on the predictive model of the TLS‐TB profile and the Differentiation of patients with CRCLM. CRCLM, colorectal cancer liver metastases; TLS, tertiary lymphoid structure; TB, tumor budding; ROC, Receiver Operating Characteristic.

**Figure 7:**
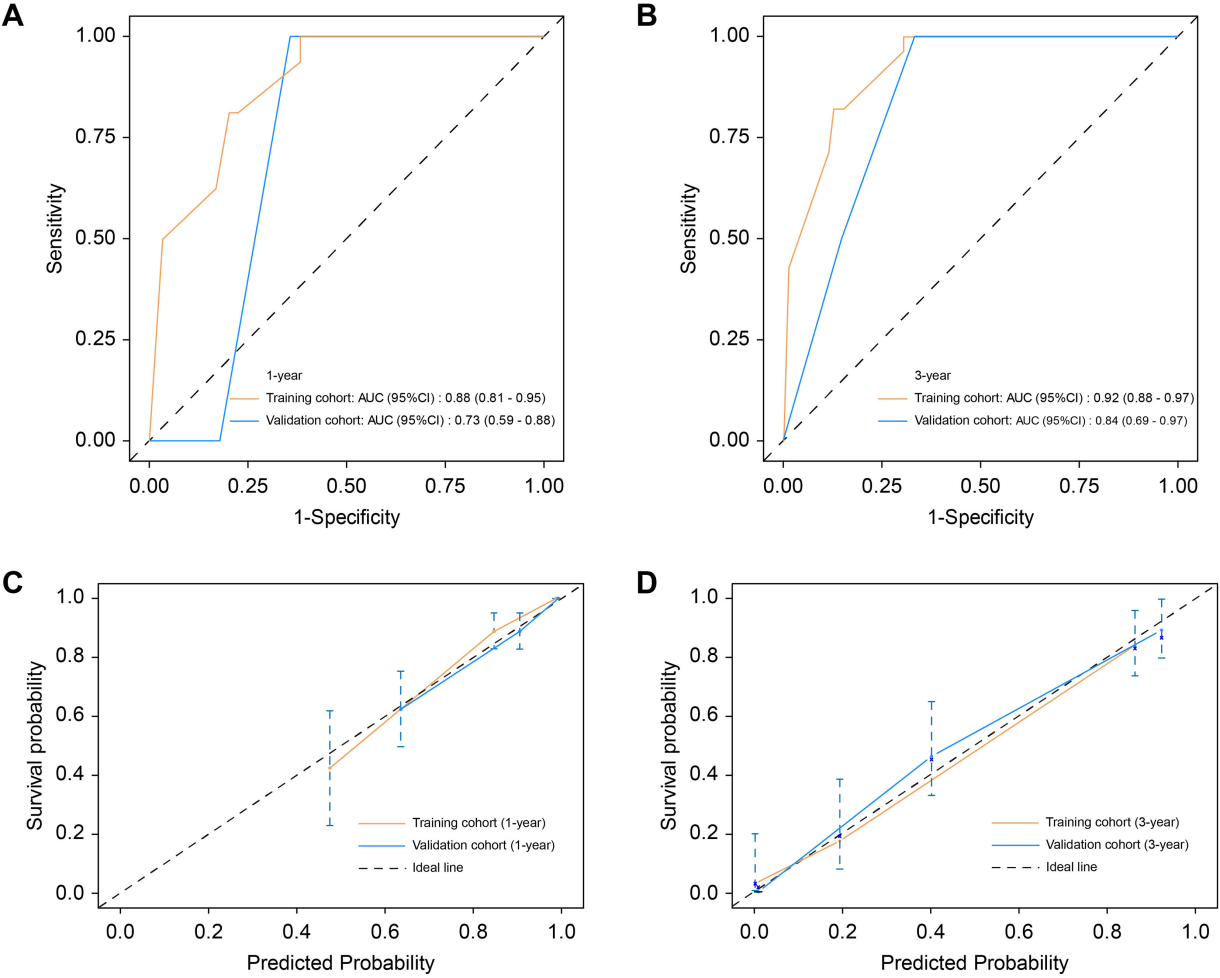
Model performance in training and validation cohorts. **(A)** The ROC curve of 1-year survival probability in CRCLM, comparing the training cohort with validation cohort. **(B)** The ROC curve of 3-year survival probability in CRCLM, comparing the training cohort with validation cohort. **(C)** Calibration curves for 1-year survival probability of CRCLM, comparing the training and validation cohorts. **(D)** Calibration curves for 3-year survival probability of CRCLM, comparing the training and validation cohorts. CRCLM, colorectal cancer liver metastases; ROC, Receiver Operating Characteristic.

Based on the results of the univariate and multivariate analyses in the training cohort, a nomogram clinical prediction model was constructed by assigning individual scores to each independent risk factor. The total score, obtained by summing these individual scores, corresponds to the model-predicted probability of overall survival (OS). Histological differentiation, TLS density, TB grade, and TLS/TB index were identified as independent risk factors for OS in patients with CRCLM. These factors were integrated into a nomogram for colorectal cancer liver metastases (**Figure 6A**). The calibration curve demonstrated excellent consistency between the nomogram-predicted outcomes and actual observations (**Figure 6B**). The nomogram was subsequently applied to the independent external validation cohort (n=30). The ROC curves for the validation cohort showed good consistency with those of the training cohort for both 1−year and 3−year survival (**Figures 7A, B**), and the calibration curves similarly demonstrated good agreement between predicted and observed survival (**Figures 7C, D**), suggesting the model’s potential generalizability and clinical utility.

### 3.5 Comparison of the accuracy and clinical utility between the two models

Although histological differentiation and the TLS/TB index demonstrate independent prognostic significance in patients with advanced colorectal cancer, it remains unclear which of these two models better predicts outcomes in patients with CRCLM. ROC curve in the training cohort analysis revealed that the TLS/TB index (AUC = 0.89) outperformed the risk stratification models based solely on histological differentiation (AUC = 0.70), TLS density (AUC = 0.80) and TB grade (AUC = 0.84) (**Figures 6C, D**), indicating superior predictive precision. Furthermore, decision curve analysis confirmed that our index provided a greater net clinical benefit compared to histological differentiation (**Figure 6E**).

## 4 Discussion

Colorectal cancer liver metastasis (CRCLM) is a highly aggressive malignancy, and current prognostic assessments rely on the integration of multidimensional biological characteristics (32). In recent years, tertiary lymphoid structures (TLS) have garnered significant attention as biomarkers of antitumor immune responses (28). This study further confirms that high TLS density correlates with prolonged overall survival (OS) (33, 34), consistent with prior research indicating that TLS enhances antitumor immunity by recruiting cytotoxic T cells and promoting TLS maturation (35, 36). We also evaluated TLS maturity using a quantitative scoring system based on germinal center formation. Although higher maturity scores were associated with better survival in univariate analysis, this effect was not independent after adjusting for TLS density, suggesting that the overall density of TLS—rather than their maturity status per se—may be the dominant prognostic factor in our synchronous CRCLM cohort. However, the tumor microenvironment is not governed by a single factor. Tumor budding (TB), a morphological manifestation of epithelial-mesenchymal transition (EMT), enhances the capacity of tumor cells to detach from the primary site and initiate metastasis. Our findings demonstrated that higher TB counts are associated with poorer patient prognosis (37). Furthermore, correlation analysis revealed an inverse relationship between TLS density and TB density. The observed inverse correlation between TLS and TB may stem from their opposing roles within the tumor microenvironment. Currently, there is a lack of research on the biological mechanisms underlying their negative correlation. We hypothesize that, on one hand, a robust TLS-mediated immune response—characterized by the production of cytokines and the activity of cytotoxic lymphocytes—may directly suppress the outgrowth and dissemination of tumor cell clusters at the invasive front, thereby inhibiting TB formation. On the other hand, an aggressive TB phenotype, driven by EMT and associated with an immunosuppressive microenvironment, may actively inhibit the recruitment and function of lymphoid cells, impairing the initiation and maturation of TLS. This dynamic seesaw between local immune surveillance and tumor invasiveness is captured by the TLS/TB index. This hypothesis requires further experimental validation.

The integration of TLS and TB into a unified index provides a hypothesis−driven integrative morphologic metric reflecting the balance between host immune organization and the invasive phenotype. Given that direct mechanistic evidence linking these morphologic features remains limited, this index serves as a surrogate tool to capture the local microenvironmental equilibrium in clinical practice. The results demonstrate that the TLS/TB index effectively stratifies patients into distinct prognostic subgroups: those with a high ratio may exhibit an “immune-active” status, while those with a low ratio tend toward an “invasion-dominant” phenotype. This model should be interpreted as a complementary prognostic tool rather than a definitive measure of underlying biological mechanisms. From a clinical translation perspective, the stratification capability of the TLS/TB index may inform personalized postoperative treatment strategies. As a hypothesis-generating observation, patients with a high TLS/TB ratio, indicative of a pre-existing, active anti-tumor immune contexture, might be hypothesized to derive greater benefit from immunotherapy. Conversely, patients with a low TLS/TB ratio, signaling dominant invasive and metastatic potential, may require intensified or novel adjuvant chemotherapy regimens targeting EMT and metastasis-initiating cells, or be considered for more frequent surveillance. It is important to note that the current cohort did not include patients treated with immunotherapy, and no response data were available. Therefore, these speculations should be interpreted with caution and require prospective validation in future studies. This model provides a rationale for future studies to test these hypotheses in clinical trials. Building upon our group’s prior findings identifying TLS as a protective factor and TB as an independent risk factor for colorectal cancer, this study introduces an integrative approach through the TLS/TB index. By quantifying this dynamic equilibrium, we developed a comprehensive model for predicting survival in patients with CRCLM. Our findings reconfirm the tumor-promoting role of TB and tumor-suppressive function of TLS. In this study, the TLS/TB index was identified as an independent prognostic factor, showing promising predictive performance not only over individual TLS density or TB grade, but also over the conventional histological differentiation parameter. These results underscore that the interplay between immune defense and invasion-metastasis mechanisms within the tumor microenvironment may play a pivotal role in determining recurrence and survival outcomes in patients with advanced cancer patients.

This study has several limitations. First, the retrospective multi-center design, while valuable, may introduce selection bias, necessitating further validation of the model’s generalizability through prospective multicenter cohorts. Second, the current quantification of TLS and TB relies on semi-quantitative pathological assessment, which could be enhanced by integrating digital pathology and artificial intelligence algorithms to improve standardization. Third, TLS were quantified based on density without subclassification by maturity (e.g., presence of germinal centers) or spatial localization (intratumoral vs. peritumoral). Given that TLS maturity has been associated with differential immune functions and prognostic implications, this represents a limitation that may affect biological interpretation. Forth, the TLS/TB index cannot fully represent the complex interactions among myriad components within the tumor microenvironment, with the roles of other elements such as fibroblasts, endothelial cells, and additional immune cells requiring further investigation. Fifth, while TB was categorized according to standardized ITBCC criteria, TLS density and the TLS/TB index were dichotomized using median cutoffs in the absence of established clinical thresholds, a limitation that future studies with larger cohorts may address by identifying optimal cutoffs. Additionally, while we developed a quantitative TLS maturity score, its lack of independent prognostic significance may be attributable to sample size limitations. Future studies with larger cohorts are needed to clarify the relative contributions of TLS density versus maturity in CRCLM.

In summary, the TLS/TB index provides a novel perspective for prognostic prediction of CRCLM by integrating bidirectional regulatory information along the “immunity-invasion” axis. Future research should focus on exploring the associations between this index and genomic features (e.g., microsatellite instability), as well as treatment responses to facilitate its translation into clinical practice.

## 5 Conclusion

These results demonstrate that the TLS/TB index serves as an integrative prognostic metric that reflects the balance between immune organization and invasive phenotype in CRCLM. This model may facilitate more granular risk stratification when used in conjunction with conventional clinicopathological parameters. After adjustment for established clinical covariates, the index remained independently associated with patient outcomes. External validation provided preliminary support for its predictive accuracy and generalizability, and its performance compared favorably with that of conventional prognostic parameters in this cohort. The integration of this index into clinical models may enhance risk stratification and facilitate personalized therapeutic strategies for patients with synchronous CRCLM.

## Data Availability

The original contributions presented in the study are included in the article/Supplementary Material. Further inquiries can be directed to the corresponding authors.

## References

[1] Bray F Laversanne M Sung H Ferlay J Siegel RL Soerjomataram I . Global cancer statistics 2022: Globocan estimates of incidence and mortality worldwide for 36 cancers in 185 countries. CA: A Cancer J For Clin. (2024) 74:229–63. doi: 10.3322/caac.21834. PMID: 38572751

[2] Shin AE Giancotti FG Rustgi AK . Metastatic colorectal cancer: Mechanisms and emerging therapeutics. Trends Pharmacol Sci. (2023) 44:222–36. doi: 10.1016/j.tips.2023.01.003. PMID: 36828759 PMC10365888

[3] Leiphrakpam PD Newton R Anaya DA Are C . Evolution and current trends in the management of colorectal cancer liver metastasis. Minerva Surg. (2024) 79:455–69. doi: 10.23736/s2724-5691.24.10363-2. PMID: 38953758

[4] Capdevila J Saura C Macarulla T Casado E Ramos FJ Tabernero J . Monoclonal antibodies in the treatment of advanced colorectal cancer. Eur J Surg Oncol (EJSO). (2007) 33:S24–34. doi: 10.1016/j.ejso.2007.09.025. PMID: 17981431

[5] Jin Z Li Y Yi H Wang M Wang C Du S . Pathogenetic development, diagnosis and clinical therapeutic approaches for liver metastasis from colorectal cancer (review). Int J Oncol. (2025) 66. doi: 10.3892/ijo.2025.5728. PMID: 39950314 PMC11844340

[6] Calderon Novoa F Ardiles V de Santibañes E Pekolj J Goransky J Mazza O . Pushing the limits of surgical resection in colorectal liver metastasis: How far can we go? Cancers. (2023) 15. doi: 10.3390/cancers15072113. PMID: 37046774 PMC10093442

[7] Chen Y Liang Z Lai M . Targeting the devil: Strategies against cancer-associated fibroblasts in colorectal cancer. Trans Res. (2024) 270:81–93. doi: 10.1016/j.trsl.2024.04.003. PMID: 38614213

[8] Wu K Zhang G Shen C Zhu L Yu C Sartorius K . Role of T cells in liver metastasis. Cell Death Dis. (2024) 15:341. doi: 10.1038/s41419-024-06726-2. PMID: 38755133 PMC11099083

[9] Xiao Y Yu D . Tumor microenvironment as a therapeutic target in cancer. Pharmacol Ther. (2021) 221:107753. doi: 10.1016/j.pharmthera.2020.107753. PMID: 33259885 PMC8084948

[10] Morabito M Thibodot P Gigandet A Compagnon P Toso C Berishvili E . Liver extracellular matrix in colorectal liver metastasis. Cancers. (2025) 17. doi: 10.3390/cancers17060953. PMID: 40149289 PMC11939972

[11] Zhou S-N Pan W-T Pan M-X Luo Q-Y Zhang L Lin J-Z . Comparison of immune microenvironment between colon and liver metastatic tissue in colon cancer patients with liver metastasis. Digestive Dis Sci. (2020) 66:474–82. doi: 10.1007/s10620-020-06203-8. PMID: 32193860

[12] Khan SU Fatima K Malik F Kalkavan H Wani A . Cancer metastasis: Molecular mechanisms and clinical perspectives. Pharmacol Ther. (2023) 250:108522. doi: 10.1016/j.pharmthera.2023.108522. PMID: 37661054

[13] Huang L-B Yang T-H Chen H-N . Predictive role of tumor budding in T1 colorectal cancer lymph node metastasis. Gastroenterology. (2021) 161:732–3. doi: 10.1053/j.gastro.2020.12.053. PMID: 33387517

[14] Lugli A Zlobec I Berger MD Kirsch R Nagtegaal ID . Tumour budding in solid cancers. Nat Rev Clin Oncol. (2020) 18:101–15. doi: 10.1038/s41571-020-0422-y. PMID: 32901132

[15] Mao Y Wang X Xi L Dong M Song P Miao J . Prediction values of tertiary lymphoid structures in the prognosis of patients with left- and right-sided colon cancer: A multicenter propensity score-matched study. Int J Surg. (2023) 109:2344–58. doi: 10.1097/js9.0000000000000483. PMID: 37247038 PMC10442147

[16] Hatthakarnkul P Quinn Jean A Ammar A Lynch G Van Wyk H McMillan Donald C . Molecular mechanisms of tumour budding and its association with microenvironment in colorectal cancer. Clin Sci. (2022) 136:521–35. doi: 10.1042/cs20210886. PMID: 35445707

[17] Qu Q Wu D Li Z Yin H . Tumor budding and the prognosis of patients with metastatic colorectal cancer: A meta-analysis. Int J Colorectal Dis. (2023) 38:141. doi: 10.1007/s00384-023-04423-8. PMID: 37222838

[18] Yang F Yang J Wu M Chen C Chu X . Tertiary lymphoid structures: New immunotherapy biomarker. Front Immunol. (2024) 15:1394505. doi: 10.3389/fimmu.2024.1394505. PMID: 39026662 PMC11254617

[19] Zhao L Jin S Wang S Zhang Z Wang X Chen Z . Tertiary lymphoid structures in diseases: Immune mechanisms and therapeutic advances. Signal Transduct Target Ther. (2024) 9:225. doi: 10.1038/s41392-024-01947-5. PMID: 39198425 PMC11358547

[20] Zhang C Wang X-Y Zuo J-L Wang X-F Feng X-W Zhang B . Localization and density of tertiary lymphoid structures associate with molecular subtype and clinical outcome in colorectal cancer liver metastases. J Immunother Cancer. (2023) 11. doi: 10.1136/jitc-2022-006425. PMID: 36759015 PMC9923349

[21] Varghese A Hess SM Chilakapati S Conejo-Garcia JR McGray AJR Zsiros E . Tertiary lymphoid structures: Exploring opportunities to improve immunotherapy in ovarian cancer. Front Immunol. (2025) 16:1473969. doi: 10.3389/fimmu.2025.1473969. PMID: 40475770 PMC12137288

[22] Langouo Fontsa M Padonou F Willard-Gallo K . Tumor-associated tertiary lymphoid structures in cancer: Implications for immunotherapy. Expert Rev Clin Immunol. (2024) 20:839–47. doi: 10.1080/1744666x.2024.2380892. PMID: 39007892

[23] Yu A Cao M Zhang K Yang Y Ma L Zhang X . The prognostic value of the tertiary lymphoid structure in gastrointestinal cancers. Front Immunol. (2023) 14:1256355. doi: 10.3389/fimmu.2023.1256355. PMID: 37868990 PMC10590053

[24] Cui X Gu X Li D Wu P Sun N Zhang C . Tertiary lymphoid structures as a biomarker in immunotherapy and beyond: Advancing towards clinical application. Cancer Lett. (2025) 613:217491. doi: 10.1016/j.canlet.2025.217491. PMID: 39862919

[25] Peyraud F Guegan J-P Vanhersecke L Brunet M Teyssonneau D Palmieri L-J . Tertiary lymphoid structures and cancer immunotherapy: From bench to bedside. Med. (2025) 6:100546. doi: 10.1016/j.medj.2024.10.023. PMID: 39798544

[26] Wang Y Zhong X He X Hu Z Huang H Chen J . Liver metastasis from colorectal cancer: Pathogenetic development, immune landscape of the tumour microenvironment and therapeutic approaches. J Exp Clin Cancer Res. (2023) 42:177. doi: 10.1186/s13046-023-02729-7. PMID: 37480104 PMC10362774

[27] Riaz AA Ginimol M Rasha R Ahmed K Ahmed A Catrin S . Revised strengthening the reporting of cohort, cross-sectional and case-control studies in surgery (Strocss) guideline: An update for the age of artificial intelligence. Premier J Sci. (2025) 2. doi: 10.70389/PJS.100081

[28] Schumacher TN Thommen DS . Tertiary lymphoid structures in cancer. Science. (2022) 375:eabf9419. doi: 10.1126/science.abf9419. PMID: 34990248

[29] Satoh K Nimura S Aoki M Hamasaki M Koga K Iwasaki H . Tumor budding in colorectal carcinoma assessed by cytokeratin immunostaining and budding areas: Possible involvement of C-Met. Cancer Sci. (2014) 105:1487–95. doi: 10.1111/cas.12530. PMID: 25220207 PMC4462370

[30] Lugli A Kirsch R Ajioka Y Bosman F Cathomas G Dawson H . Recommendations for reporting tumor budding in colorectal cancer based on the International Tumor Budding Consensus Conference (Itbcc) 2016. Mod Pathol. (2017) 30:1299–311. doi: 10.1038/modpathol.2017.46. PMID: 28548122

[31] Marcoulides KM Raykov T . Evaluation of variance inflation factors in regression models using latent variable modeling methods. Educ Psychol Meas. (2019) 79:874–82. doi: 10.1177/0013164418817803. PMID: 31488917 PMC6713981

[32] Al Bandar MH Kim NK . Current status and future perspectives on treatment of liver metastasis in colorectal cancer. Oncol Rep. (2017) 37:2553–64. doi: 10.3892/or.2017.5531. PMID: 28350137

[33] Tokunaga R Nakagawa S Sakamoto Y Nakamura K Naseem M Izumi D . 12‐Chemokine signature, a predictor of tumor recurrence in colorectal cancer. Int J Cancer. (2020) 147:532–41. doi: 10.1002/ijc.32982. PMID: 32191346 PMC9371443

[34] Chen X Wu P Liu Z Li T Wu J Zeng Z . Tertiary lymphoid structures and their therapeutic implications in cancer. Cell Oncol. (2024) 47:1579–92. doi: 10.1007/s13402-024-00975-1. PMID: 39133439 PMC12974082

[35] Fridman WH Sibéril S Pupier G Soussan S Sautès-Fridman C . Activation of B cells in tertiary lymphoid structures in cancer: Anti-tumor or anti-self? Semin Immunol. (2023) 65:101703. doi: 10.1016/j.smim.2022.101703. PMID: 36481358

[36] Vion R Roulleaux-Dugage M Flippot R Ouali K Rouanne M Clatot F . Induction of tertiary lymphoid structures in tumor microenvironment to improve anti-tumoral immune checkpoint blockade efficacy. Eur J Cancer. (2025) 225:115572. doi: 10.1016/j.ejca.2025.115572. PMID: 40517527

[37] Grigore A Jolly M Jia D Farach-Carson M Levine H . Tumor budding: The name is Emt. Partial Emt. J Clin Med. (2016) 5. doi: 10.3390/jcm5050051. PMID: 27136592 PMC4882480

